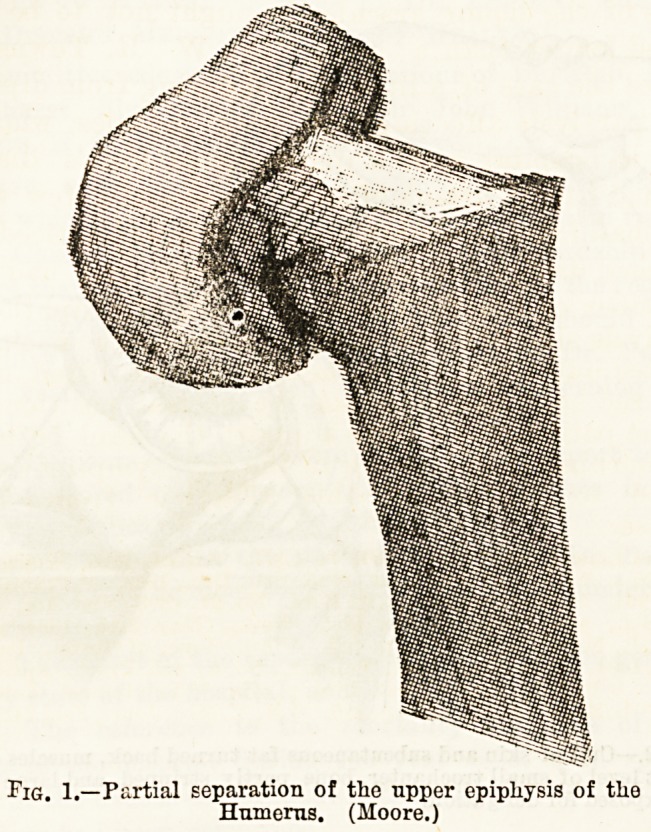# Bone, Joint, and Orthopædic Surgery

**Published:** 1894-07-28

**Authors:** 


### PROGRESS IN SURGERY.

### BONE, JOINT, AND ORTHOPÆDIC SURGERY.

### Dislocations.

**Treatment of Recurrent Dislocations.**—Dr. Ricard attributes the recurrence of a certain number of dislocations of the shoulder to the relaxation of the articular capsule, which in consequence of the initial dislocation remains depressed in the form of a *cul-de-sac*, so that on the least motion being made by the patient, the head of the humerus leaves the glenoid cavity and lodges in the capsular sac. To remedy this, Ricard has sutured the sac on two occasions with success. The limb is kept immobile for about five weeks, as it is maintained that immobility is the best means of preventing relaxation of the capsule. A similar condition of recurring dislocation in the knee has been dealt with by Le Dentu.[2] The case was that of a little girl, aged seven, in whom a fall had determined abnormal mobility of the patella to such an extent that whenever she made a mis-step a complete external dislocation of this bone took place. After a while, flexion of the leg on the thigh sufficed to displace the patella, and caused the child to fall. To remedy this condition, Le Dentu exposed the capsule and took up the exuberant portion in a large vertical fold by means of sutures. The result is said to have been excellent, as the dislocation never recurred.

**Late Reduction of Dislocated Inferior Maxilla.**—C. A. Powers[3] describes the case of a young woman whose jaw slipped out apparently during sleep. At the end of 136 days she was brought to Powers. Under the influence of ether, a speculum was placed between the molar teeth, and the jaw forced open by a piece of wood. By manipulation extending over twenty minutes, the dislocation was finally reduced. The lower incisors still rested a little in front of the upper, due apparently to slight thickening in the glenoid fossa. A plaster of Paris bandage was applied and left on for four weeks, the patient being fed meanwhile through the nose. After removal of the splint, restoration of function commenced and is now complete. The luxation has not recurred; hitherto the longest period recorded was 100 days.

**Dislocation of the Femur.**—Christopher Heath[4], in a clinical lecture at University College Hospital, commented on the comparative rarity of luxation occurring at this joint. During a period of twelve years, only 17 cases were seen. He quotes Malgaigne, who states that of 491 cases of dislocation in L'Hotel Dieu 34 only occurred in the hip. Heath inclines to Eve's opinion, in opposition to Morris's, that all dislocations do not take place primarily through the lower and back parts of the capsule, but that dorsal dislocations take place directly through the upper and posterior part of the capsule. In the case of the man commented on, reduction was effected by Heath on the 106th day. Riedinger[5] states that the methods usually employed in determining the amount of lengthening or shortening of the limbs in cases of hip-joint dislocations give erroneous results. He claims that the measurement taken from the anterior superior spine of the ilium will show less alteration than if the measurement be taken from the middle line of the body. It is recommended, therefore, in such cases to take the measurement from the symphysis pubis. In four cases, measurements from the spine indicated shortening; but when taken from the symphysis, a lengthening, as compared with the sound side.

**On the Treatment of Old Luxations of the Shoulder.**—Thiery[6] records a case of dislocation of the shoulder of three months' standing. He advises in such instances that the method of Kocher should first be tried. If this fail, counter extension and co-aptation under ether should be attempted. If this is unsuccessful, a free

Fig. 1.—Partial separation of the upper epiphysis of the Humerus. (Moore.)

incision down to the articulation should be made, the glenoid cavity deepened, if necessary, the head of the bone freed, and placed in its new position. If, when the operation is attempted, the head of the bone is so firmly fixed in its new position that it is impossible to move it, resection of the humerus is indicated. Dr. W. Stewart[7] showed a case of compound sub-astragaloid dislocation of the foot. A seaman, aged 24, dropped 25 feet into the hold of a ship, landing on the left foot. When seen, the left astragalus was found dislocated, downwards, inwards, and forwards, and projecting through a wound on the inner side of the foot, the foot being extremely everted. Under chloroform a piece of the os calcis, measuring one inch by half an inch, was found lying loose. This was removed, and an extension being applied to the metatarsus, the parts were comparatively easily put into position. Two months afterwards, the patient was walking well without crutch or stick.

**Spring-knee.**—Delorme[8] gives particulars of the case of a patient with an affection of the knee, characterised by a sudden jerk occurring at the end of each movement of extension. A year ago he was thrown from his horse, and was dragged for about 30 feet. For four months afterwards he suffered with acute hydrarthrosis. In walking, when he endeavours to extend the right leg, a sudden jerk occurs, as if the articulation unlocked at the moment when the knee is flexed to an angle of 160 deg., and is communicated to the whole thigh; after which the movement of extension, without being at all arrested, continued at a more rapid rate. If the movement is passive or very slow it does not occur. There is no apparent change in the knee itself; the capsule is somewhat thickened, and in complete extension there is very obvious lateral mobility, and the triceps is atrophied.

In seeking for an explanation, Delorme dismisses the possibility of the crucial ligaments being ruptured, and naturally discusses the analogy of this affection with that of "trigger-finger." Both conditions he believes to be due to functional spasm, a view shared by Carlier and Verneuil. Everything speaks in its favour; the intensity of the jerk is increased by fatigue; it is sometimes followed by tremor, and the shock is transmitted to the whole thigh. It ceases completely when the muscles are paralysed by compression of the thigh with Esmarch's bandage, and it returns when the bandage is removed.

[1] Med. Week, April 6th, 1894, p. 166. [2] Med. Week., April 4th, 1894. [3] N.Y. Surg. Soc., November, 1893, p. 239. [4] Clin. Jour., December 27th, 1893, [5] Deutscher, Zeit. für. Chir. Bd. xxxvi., S. 102. [6] Ther. Gaz., March 15th, 1894, p. 200; and La Trib. Méd. 27me année, 2me Ser. No. 4. [7] Edin. Med. Jour., March, 1894, p. 827. [8] Med. Week, April 13th, 1894.

*(To be continued.)*

## Figures and Tables

**Fig. 1. f1:**